# The impact of early comprehensive geriatric screening on the readmission rate in an acute geriatric ward: a quasi-experimental study

**DOI:** 10.1186/s12877-019-1312-y

**Published:** 2019-10-24

**Authors:** Kun-Pei Lin, Jen-Hau Chen, Feng-Ping Lu, Chiung-Jung Wen, Ding-Cheng (Derrick) Chan

**Affiliations:** 1 Department of Geriatrics and Gerontology, National Taiwan University Hospital, Taipei, Taiwan; 2 Department of Internal Medicine, National Taiwan University Hospital, Taipei, Taiwan; 3 Department of Family Medicine, National Taiwan University Hospital, Taipei, Taiwan; 4 National Taiwan University Hospital Chu-Tung Branch, No. 1, Changde St., Taipei, 100 Taiwan

**Keywords:** Readmission, Comprehensive geriatric assessment, Screening

## Abstract

**Background:**

Unplanned readmission is an important healthcare quality issue. We studied the effect of a comprehensive geriatric screen (CGS) in the early admission course followed by a comprehensive geriatric assessment on readmission rates in elderly patients.

**Methods:**

This quasi-experimental study with a historical comparison group was conducted in the geriatric ward of a referral centre in northern Taiwan. Older adults (aged > = 65 y/o) admitted from June 2013 to December 2013 were recruited for the geriatric screen group (*N* = 377). Patients admitted to the same ward from July 2011 to June 2012 were selected for the historical group (*N* = 380). The CGS was administered within the first 48 h after admission and was followed by a comprehensive geriatric assessment (CGA). Confounding risk factors included age, gender, Charlson comorbidity index, Barthel index score and medical utilization (length of stay and number of admissions), which were controlled using logistic regression models. We also developed a scoring system to identify the group that would potentially benefit the most from the early CGS.

**Results:**

The 30-day readmission rate was significantly lower in the early CGS group than in the historical comparison group (11.4% vs 16.9%, *p* = 0.03). After adjusting for confounding variables, the hazard ratio of the early CGS group was 0.64 (95% CI 0.43–0.95). After scoring the potential benefit to the patients in the early CGS group, the log rank test showed a significant difference (*p* = 0.001 in the high-potential group and *p* = 0.98 in the low-potential group).

**Conclusion:**

An early CGS followed by a CGA may significantly reduce the 30-day readmission rate of elderly patients.

## Introduction

A successful or optimal admission course provides patients with relief or remission from acute disease. Therefore, unplanned readmission rates have been used as a quality indicator of medical care [1]. Frequent hospital admissions are associated with poor clinical outcomes, such as increased length of stay and increased disability [2–4].

High readmission rates also lead to economic burdens for patients and the healthcare system. The Centers for Medicare and Medicaid Services have adopted programmes such as payment penalties for hospitals with excess readmissions for several conditions [5].

However, the risk factors for patient readmission are complicated and may vary across different diseases or patient groups. For example, problems with readmission rates seem to be more severe in large hospitals, teaching hospitals and safety-net hospitals because of increased medical complexity and socioeconomic variability [6]. Previous studies have shown that the 30-day readmission rates varied from 7.3 to 32.7% [7–10]. Among the existing risk prediction models, comorbidities, illness severity, prior use of medical service, functional status, and sociodemographic factors are often cited [11].

Elderly patients seem to be more fragile, with more comorbidities and a higher readmission risk. To address this complicated problem in this susceptible group, a well-designed evaluation may be needed. The comprehensive geriatric assessment (CGA) was developed and is used for elderly patients with recent functional decline, geriatric syndrome, and multiple comorbidities, and it may have benefits regarding physical and cognitive function, the capacity to live at home, and reduced admission frequencies during study follow-up periods [12, 13].

In addition, the CGA has been applied to cancer patients to predict their mortality and prognosis [14]. It has demonstrated the possibility of CGA-guided cancer therapy [15] in the future.

Readmission is a multifactorial and complicated problem, similar to geriatric syndrome. Elderly patients with geriatric syndrome benefit from the CGA, and results indicate that it is possible to better manage elderly patients with a high risk of readmission.

However, the complete CGA takes too long to administer to all patients in daily clinical practice. Several brief forms or screening forms of the CGA have been developed, but they are mostly used for specific conditions [16–18], and there is no conclusive form for general care. We conducted a comprehensive geriatric screen (CGS) in the early admission course of patients in the acute geriatric ward to improve CGA efficiency. The aim of this study was to evaluate the impact of an early CGS on readmission rates for older adults. In addition, because of the heterogeneity of patients and limited medical resources, we also attempted to identify the population in our study that would most benefit from the CGS.

## Methods

### Setting

This study was conducted in the geriatric ward of the National Taiwan University Hospital. The geriatric ward serves elderly patients with acute medical illness requiring admission.

### Design

This quasi-experimental prospective study included a historical comparison group and a post-intervention group.

A total of 415 patients admitted from July 2011 to June 2012 were included in the historical comparison group. They were randomized selected from the 848 patients admitted to geriatric ward in a one-year period. The CGA was performed on selected frail inpatients (in 40 of 415 patients, about 9.6% of controls). We had reviewed the medical chart and confirmed the patients discharged alive. The same patient would be enrolled once, even if he or she had admission several times in this year. We also check the patients’ medical chart at outpatient department (OPD) within 2 months after discharge. If the patient loss follow up at OPD in this period would label as censored case.

Patients admitted from June 2013 to December 2013 were recruited for the CGS group (*N* = 409) and received the CGS evaluation within the first 48 h after admission. The CGA was performed for selected patients with positive CGS results.

All patients were elderly (age > =65) at admission and under routine geriatric medical care, including functional evaluations, disease treatments and care-skill educations.

Participants were excluded if they died prior to discharge or were transferred to another medical department. In the CGS group, written informed consent was obtained from participants or from their legal guardians if the patients had serious cognitive impairment. The study was approved by the Institutional Review Board of the National Taiwan University Hospital (No: 201303058RINC).

### Measures

Data collected from medical charts included demographic data, disease comorbidities, functional status and health utilization. Participants in the CGS group received the CGS within the first 2 days of admission. The CGS used simplified but valid screening tools, e.g., the Confusion Assessment Method (CAM) criteria for delirium [19], the core symptoms for depression [20], and the Mini-Cog for dementia [21]. It also maintained the core metrics of the CGA, which consisted of measures of delirium, depression, dementia, visual and hearing capacity, physical performance, falls, polypharmacy, pain, pressure sores, incontinence, tubes, nutrition, caregiver issues, and socioeconomic issues. The CGS was administered by the same study nurse who received geriatric skill training before the study, and she accompanied our experienced nurse practitioners during the first 2 weeks of practice. The entire evaluation took approximately 10–15 min depending on the patient’s condition. All the results were provided to the attending physician of the geriatric ward. If the patient had positive CGS results, a complete CGA was conducted for further evaluation and management (Additional file 1: Table S1). Patients received non-pharmacologic or pharmacologic management, rehabilitation, nutrition modification, fall prevention and care-skill education according to their needs and the physician’s suggestion.

### Definition of readmission

All patients were followed for 30 days after hospital discharge by medical chart review and/or telephone calls. The main outcome was unexpected patient readmission within 30 days after discharge. Planned readmissions, such as admissions for cancer chemotherapy, scheduled operations or scheduled rehabilitation, were defined as scheduled admissions and were not included as part of the main outcome. Patients who died within 30 days at home or in an emergency room after discharge were excluded from the analysis.

### Statistical analyses

According to previous admission data, the required sample size was calculated before the study to obtain 80% power at the 5% significance level [22].

T-tests (for normally distributed continuous variables) and chi-square tests (for categorical variables) were used for intergroup comparisons.

Curves for the probability of no readmission within 30 days were created with the Kaplan–Meier method and compared using the log-rank test. A multivariate analysis for possible significant factors was performed using the Cox proportional regression model. We developed several models to test the stabilization of the effect of CGS.

Stratified analyses were performed using age, gender, the Charlson comorbidity index (CCI), the Barthel index score, and number of admissions within 1 year to assess the association between the CGS and the 30-day readmission risk.

Finally, we used a scoring system to identify the patients who received the most benefit from the CGS. The Kaplan–Meier method and the log-rank test were used to analyse the differences between the scores.

All statistical tests were two-sided. A *p*-value < 0.05 was considered statistically significant. SAS version 9.4 (SAS Institute, Cary, NC) was used for statistical analyses.

## Results

### Demographics

**Fig. 1:**
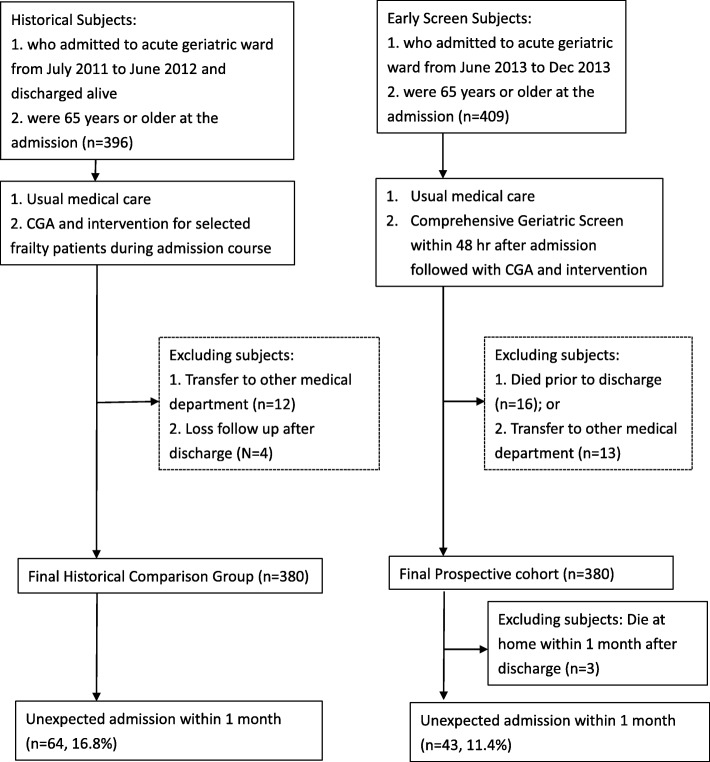
Study flowchart. Historical control (left) and early Comprehensive Geriatric Screen group (right)

After excluding patients who died during the study course or were transferred to other medical departments, a total of 380 controls and 377 patients who received the CGS were included in our final cohort study (Fig. 1). There were 9 patients with scheduled admissions (3 patients in the control group and 6 patients in the CGS group) within 30 days of discharge. The number of scheduled admissions in the two groups was not significantly different (*p* = 0.50), and all of them were censored at their scheduled admission date. Three of the patients in the CGS group died at home or in an emergency room before readmission within 30 days of discharge and were excluded from the final analysis.

The CGS group had fewer patients with a history of hypertension and dementia but more patients with a history of stroke than the historical group. Age, gender, other comorbidities, and functional status were similar between the two groups. The 30-day readmission rate was significantly lower in the CGS group than in the historical group (11.4% vs 16.9%, *p* = 0.03, Table 1).

**Table 1:** Demographic, clinical characteristics, and functional status of the comprehensive geriatric screen (CGS) group and historical control group

	CGS group [*N* = 377]	Control group [*N* = 380]	*p* value
Men, no.(%)	191 (50.7)	202 (53.2)	0.49
Age, years, mean (SD)	81.9 ± 8.6	82.1 ± 8.1	0.77
Weight, Kg, mean (SD)	58.9 ± 12.1 [*N* = 338]	57.3 ± 13.1 [*N* = 349]	0.09
Marital status, no.(%)			
Divorced, widow, or single	165 (43.8)	167 (43.2)	0.87
Education, no.(%)			
College/University and above	47 (12.5)	43 (11.3)	0.62
Comorbidities, no.(%)			
Hypertension	260 (69.0)	290 (76.3)	0.02
Diabetes mellitus	155 (40.8)	123 (32.4)	0.02
Stroke history	154 (40.9)	126 (33.2)	0.03
Coronary artery disease	86 (22.8)	67 (17.6)	0.08
Chronic obstructive pulmonary disease	63 (16.7)	50 (13.2)	0.17
Congestive heart failure	67 (17.7)	71 (18.7)	0.75
Atrial fibrillation	46 (12.2)	54 (14.2)	0.41
Dementia	89 (23.6)	117 (30.8)	0.03
Peripheral artery disease	35 (9.3)	26 (6.8)	0.22
History of peptic ulcer	61 (16.2)	75 (19.7)	0.20
Chronic liver disease	36 (9.6)	31 (8.2)	0.45
Connective tissue disease	6 (1.6)	5 (1.3)	0.76
Chronic kidney disease	54 (14.3)	65 (17.1)	0.29
Tumor history	53 (14.1)	69 (18.3)	0.12
Metastatic cancer	10 (2.7)	7 (1.8)	0.45
Charlson comorbidity index ≥3	188 (49.9)	192 (50.5)	0.86
Barthel index score at admission ≤35	213 (56.5)	202 (53.2)	0.36
No. of admission^a^, no.(%)			0.06
0	209 (55.4)	242 (63.7)	
1–3	149 (39.5)	125 (32.9)	
4+	19 (5.0)	13 (3.4)	
Length of stay ≥10 days, no.(%)	185 (48.7)	210 (55.3)	0.07
Scheduled re-admission	6 (1.6)	3 (0.8)	0.50
30 days re-admission	43 (11.4)	64 (16.9)	0.03

^a^Within 1 year before admission date

We also examined the characteristics of the patients who were readmitted within 30 days and compared them with those who were not (Additional file 2: Table S2). Patients who were readmitted tended to have a longer lengths of stay, more comorbidities, poorer functional status at admission (Barthel index score ≤ 35), and more prior admissions within the previous year than patients who were not readmitted. Regarding specific diseases, patients with congestive heart failure, atrial fibrillation, history of peptic ulcer, chronic kidney disease, and metastatic cancer were at a higher risk of 30-day readmission.

### Main model

**Fig. 2:**
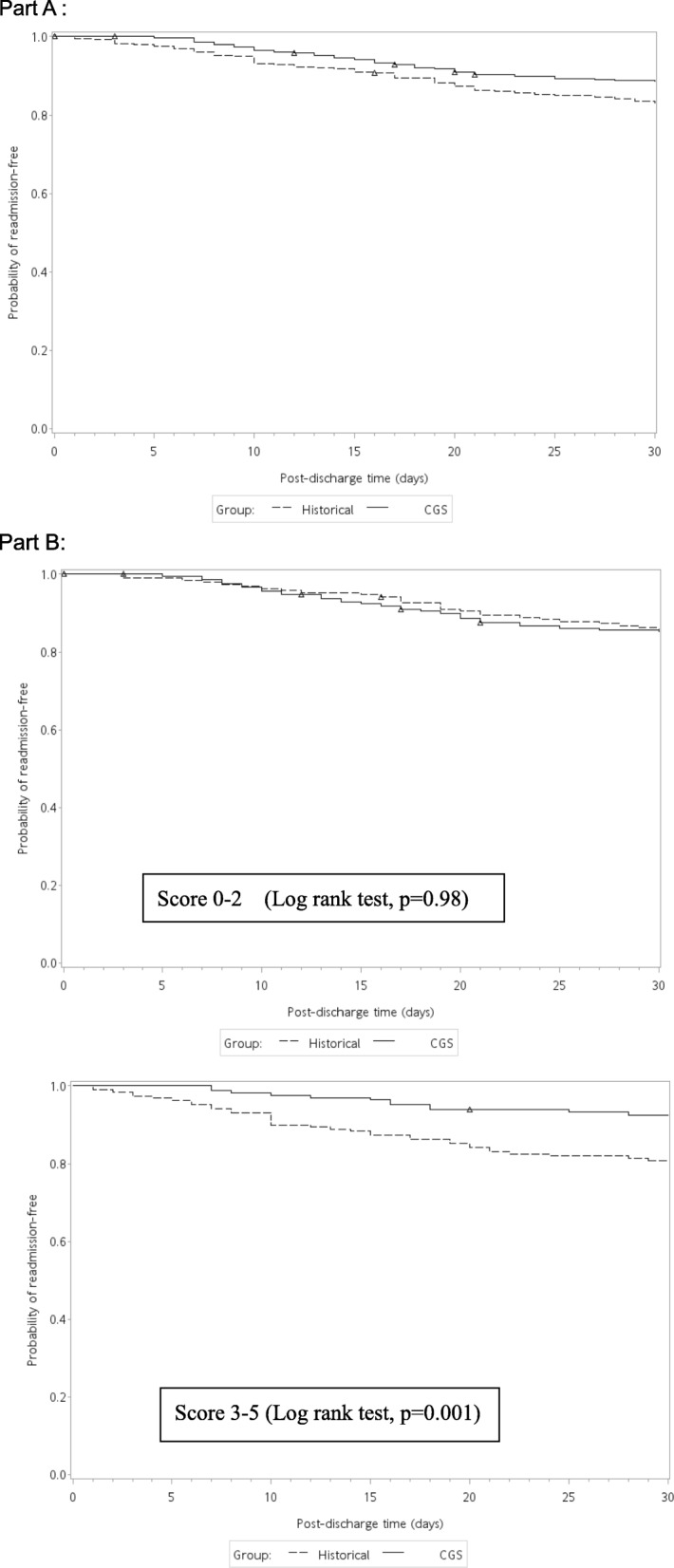
Part **a**: The probability of no readmission within 30 days in the CGS group and historical control. Δ:censored case. Part **b**: The probability of no readmission in the scoring system. Scoring system: Any variable including age < =80, women, CCI > =3, BI<=35, no admission prior 1 year. Δ:censored case

The probability of being readmission-free within 30 days of discharge between the CGS and historical groups was analysed using the Kaplan–Meier method (Fig. 2a), and the *p*-value by the log-rank test was 0.03. Before the proportional hazards analysis, we tested the time interaction model between the time variable and study groups with no significant finding (*p* = 0.60). Different models were developed to confirm the effect of the CGS (Table 2). We adjusted age and gender in model 1, and we added the disease factors (Charlson comorbidity index), functional status (Barthel index score) and medical utilization (length of stay and number of admissions) separately to the analysis. Model 5 was a completed, adjusted model for the above factors. The hazard ratio of the CGS for the different models ranged from 0.64 (95% CI 0.43–0.94 in model 4 and 0.43–0.95 in model 5) to 0.67 (95% CI: 0.45–0.98 in model 1 and 0.45–0.98 in model 3). In addition, several factors had significant effects on our readmission model. A higher Charlson comorbidity index resulted in a higher readmission risk in model 2 (HR: 1.85 95% CI: 1.24–2.75) and model 5 (HR: 1.64 95% CI: 1.09–2.46). Additionally, patients with a lower Barthel index score at admission, greater length of hospital stay or prior admission history within 1 year had a higher risk of readmission (Table 2).

**Table 2:** Hazzard ratio of various models for 30-days re-admission

	Model 1	Model 2	Model 3	Model 4	Mode1 5
Age	1.01 (0.99–1.03)	1.01 (0.98–1.02)	1.00 (0.98–1.03)	1.01 (0.98–1.03)	1.00 (0.98–1.02)
Male gender	1.39 (0.95–2.05)	1.35 (0.92–1.99)	1.38 (0.94–2.04)	1.44 (0.97–2.12)	1.41 (0.95–2.08)
Early comprehensive geriatric screen	0.66* (0.45–0.98)	0.66* (0.45–0.97)	0.67* (0.45–0.98)	0.64* (0.43–0.94)	0.64* (0.43–0.95)
Charlson comorbidity index ≥3		1.85* (1.24–2.75)			1.64* (1.09–2.46)
Barthel index score at admission ≤35			1.6* (1.07–2.37)		1.23 (0.80–1.90)
Length of stay ≥10 days				1.53* (1.03–2.27)	1.42 (0.95–2.15)
No. of admission^a^,					
1–3 vs 0				1.3 (0.88–1.98)	1.16 (0.76–1.77)
4+ vs 0				3.6* (1.84–6.87)	2.92* (1.49–5.74)

^*^p < 0.05

^a^Within 1 year before admission date

### Stratification

Stratification with key variables was important to identify the benefit of an early CGS for readmission. Age, gender, CCI, Barthel index score, and number of admissions within the prior year were chosen for stratification. The model was fully adjusted except for the stratification variables, and the results are shown in Table 3. The CGS had a significant benefit for patients <=80 years of age (HR: 0.35), women (HR: 0.51), those with a Barthel index score < =35 (HR: 0.53), and no admission history within the prior year (HR: 0.52). In the crude and basic models, the CGS showed benefits for patients with high comorbidity (CCI > =3), but this was not the case in the fully adjusted model.

**Table 3:** Comparison of 30-day readmissions between historical and the CGS group

	30-day readmission		
	Historical group Readmission/Total	CGS group Readmission/Total	HR (95% CI)
Age			
> 80 years old	42/236	32/223	0.78 (0.49–1.25)
< =80 years old	22/144	11/154	0.36 (0.17–0.76)*
Gender			
Men	36/202	28/191	0.74 (0.45–1.23)
Women	28/178	15/186	0.51 (0.27–0.97)*
Charlson Comobidity index			
< 3	23/188	15/189	0.65 (0.34–1.24)
> =3	41/192	28/188	0.66 (0.41–1.08)
Barthel index score			
> 35	40/178	20/164	0.82 (0.46–1.48)
< =35	24/202	23/213	0.53 (0.31–0.90)*
Admission times within 1 year			
> =1	27/138	27/167	0.85 (0.49–1.45)
0	37/242	16/210	0.52 (0.29–0.94)*

^*^p < 0.05

All models were adjusted for age, gender, length of stay, comorbidity, Barthel index score, admission times except stratification variable

*Abbreviations*: *CGS* comprehensive geriatric screen, *CI* confidence interval

### Scoring system

Based on the stratification variables, we scored the potential benefit to patients using the following factors: age < =80, female gender, CCI > =3, BI<=35, and no admissions within the prior year. We divided the patients into 2 groups, a low-potential group with individual scores of 0–2 and a high-potential group with scores of 3–5. The results of the two groups were analysed using the Kaplan–Meier method (Fig. 2b), and the log-rank test showed that the *p*-value was 0.98 in the low-potential group and 0.001 in the high-potential group.

## Discussion

In our study, the early CGS followed with a CGA resulted in significantly improved 30-day readmission rates in an acute geriatric ward when controlling for other risk factors. Patients who were under age 80, female, had more comorbidities, had greater functional impairment, or who had already been admitted within the past year seemed to benefit more from the early CGS.

Although some readmissions may be prevented, the composition of this avoidable proportion remains unclear [23]. The 30-day readmission rate in our control group was 16.9%, which was similar to the result of a previous study in a tertiary referral centre [24]. Several prediction models for readmission have been reported previously; some were designed for the general population, and some were limited to specific diseases, e.g., heart failure, acute myocardial infarction or pneumonia [25, 26]. These models may help physicians identify patients with a high risk of readmission, but most of the underlying risk factors, such as disease severity, comorbidities, length of stay, and previous medical utilization, cannot be changed during admission [8]. In addition, a single intervention usually had a limited ability to reduce the readmission rate [27]. Multifaceted interventions have been effective in some reports [28, 29], but the complexity of these interventions has restricted their clinical use [30].

Based on a previous report, we attempted to develop an early evaluation system that combined a multifaceted approach with ease of use to help solve the readmission problem. Combined with the use of electronic medical records, it took an average of approximately 10–15 min to perform the CGS, which was less time than the complete CGA required in each case. This multifaceted evaluation and screening tool identified those patients initially at risk for readmission. Experienced geriatricians could then easily continue with the full CGA or develop a treatment plan after receiving the CGS results.

As above statement, there are many differences between our CGS method and the origin method we used CGA in the ward (historical control group). Although patients in both groups may have a chance to receive CGA, the key difference between this two groups was time point and proportion of patients receiving CGA (Additional file 3: Table S3). Because CGA takes a lot time to administer, only about 10% of admission patients received CGA in control group (usually administered in patients with frailty and recent functional decline). In addition, CGS conducted within first 48 h after admission gives physicians plenty of time to manage geriatric problems in whole hospital course.

Consequently, our study reports a new model of care based on the use of the CGS in an acute geriatric ward. The CGS is more time efficient and preserves the ability of the traditional CGA in decreasing readmission rates, as shown in previous studies [28, 31].

There were several groups identified in this study who benefited more from the CGS. This may be because patients with a high readmission risk are heterogeneous, and not all readmissions are preventable. The CGS was more effective for patients under 80 years of age than for older patients, which may be due to their relatively good rehabilitation potential and recovery ability. Women were also good candidates for the CGS. Although the definitive reason is unclear, there are several studies that discuss sexual dichotomies in disease treatment and outcomes [32, 33]. Women were found to receive less aggressive management for some diseases than men [34], although they seemed to have more risk factors than men. Additionally, it is possible that the CGS may have documented a disease or geriatric condition before an irreversible functional decline.

Considering the clinical implications, the specific diagnosis and functional status of the patient are the most discussed factors in previous reports. Although multiple comorbidities and poor physical function at admission have been associated with a high readmission rate [7, 35, 36], patients who received the CGS seemed to reverse this trend, as these two factors were associated with a significant reduction in the readmission rate in our study. Like the CGA, the CGS may have limited benefits for patients with simple diseases or good baseline functional status. In contrast, as multifaceted tools, the CGA and CGS provide a comprehensive evaluation and can address complex conditions, such as recent functional decline, geriatric syndrome or multiple comorbidities, all of which are difficult to manage with the traditional disease treatment model.

Moreover, the CGS was beneficial to patients presenting with their first admission within the past year than in patients with recurrent admissions. There are two possible explanations for this that are worth exploring. First, patients at their first admission usually have a relatively good functional status and may recover well after appropriate management and care. Second, in patients with recurrent admissions, some preventable causes of readmission may be resolved during previous admissions, which would partially reduce the effect of the CGS, especially if the prior admission occurred in a geriatric ward.

The length of hospital stay (LOS) was also an important factor for predicting readmission risk, and this association has been reported in several studies [8, 24]. Long admission courses reflect a higher risk of readmission. Our study included this factor in the model but did not include it in our potential benefit scoring system. This was because the CGS should be performed within 48 h after admission, and the LOS is still unknown at that time; however, we found that the CGS significantly benefited patients with longer LOSs (≥10 days) compared to shorter LOSs (data not shown).

To the best of our knowledge, this is the first study to use the CGS, or a brief form of the CGA, with readmission as the primary outcome to be analysed. We had fixed attending physicians and a fixed ward setting for the duration of the study, which may have avoided some of the confounding factors associated with different care policies. Instead of quality or plan descriptions, the study provided objective data to show the exact improvement in 30-day readmission rates. The CGS can be performed easily in clinical practice early in the admission course. The scoring system for groups who could potentially benefit amplified the effect of the CGS and changed the readmission trend in the high-risk group.

There were some limitations to our study. First, our study was performed in a geriatric ward of a tertiary medical centre, and the CGS results were documented and interpreted by a geriatrician. The external validity may thus be limited. Second, the study design lacked randomization, which led to the heterogeneity of the patients’ conditions and disease types. Third, there were no definite control groups since some of the patients have received a CGA although the time point was later and the proportion of patients receiving CGA was lower than CGS group. Consequently, this study was a quasi-experimental study. At last, due to lack of intervention data (physiotherapy, nutrition ...) in historical control group, the comparison of the interventions between the two groups is difficult. Therefore, the effect resulting from difference of intervention was unknown.

## Conclusion

The CGS, as a brief CGA variant or screening form, is an initial and effective screening tool. Early CGS follow by CGA and further management may improve the 30-day readmission rate in an acute geriatric ward. Appropriately selecting patients for CGS according to age, gender, comorbidities, functional status and previous admission history may yield the most benefit in terms of readmission reduction with limited medical resources.

## Supplementary information

**Additional file 1: Table S1.** Comprehensive geriatric screen and further plan. Subjects and criteria for CGS and the method of follow-up or management.

**Additional file 2: Table S2.** Comparison of the readmission vs Non-readmission group. The characteristics of the patients who were readmitted within 30-days compared with those who were not.

**Additional file 3: Table S3.** Difference of criteria between historical control group and CGS group. The same and difference criteria between historical control group and CGS group (inclusion criteria, exclusion criteria, CGS and CGA).

## Data Availability

The datasets used and/or analysed during the current study are available from the corresponding author upon reasonable request.

## Abbreviations

CAM: Confusion Assessment Method
CCI: Charlson comorbidity index
CGA: Comprehensive geriatric assessment
CGS: Comprehensive geriatric screen
LOS: Length of hospital stay
