# Supplementary material for: The impact of early comprehensive geriatric screening on the readmission rate in an acute geriatric ward: a quasi-experimental study
Source: BMC Geriatr. 2019 Oct 24;19:285. doi: 10.1186/s12877-019-1312-y (PMC6813968; doi:10.1186/s12877-019-1312-y)
Supplement: Supplementary file 1 — Additional file 1: Table S1. Comprehensive geriatric screen and further plan. Subjects and criteria for CGS and the method of follow-up or management. [file 12877_2019_1312_MOESM1_ESM.docx]

Additional file 1: TableS1: Comprehensive geriatric screen and further plan

| Problem list | CGS Evaluation | Further plan or management |
| --- | --- | --- |
| Delirium | Confusion Assessment Method (CAM):  ( ) 1. Acute onset with fluctuation  ( ) 2. Inattention  ( ) 3. Disorganized thinking  ( ) 4. Altered level of consciousness  **Positive: 1 & 2 + (3 or 4)** | Cause evaluation.  Nonpharmacologic/pharmacologic management |
| Depression | ( ) Depressed mood  ( ) Loss of interest  ( ) Suicidal ideation (within 2 weeks)  **Positive: any of the above** | GDS  Depression management and follow up |
| Dementia | Mini-Cog: (correct: V; incorrect: X)  1. 3-item registration: ( )腳踏車 ( )紅色 ( )快樂  2. Clock drawing test: ( )  3. 3-item recall: ( )腳踏車 ( )紅色 ( )快樂  **Positive: 3-item recall = 0 or**  **3-item recall: 1-2 with abnormal CDT** | MMSE  Dementia workup  Dementia care education |
| Eyes | Uncorrected visual / hearing impairment affects daily activity  **Positive: any of the above** | Caregiver education  Fall prevention  Consult Ophthalmologist /ENT if indicated |
| Ears |  |  |
| Physical performance | Functional decline within 1 year  ( ) ADL ( ) IADL  ( ) Gait / balance  **Positive: any of the above** | Fall prevention  Neurologic exam  Rehabilitation (PT/OT) |
| Falls | ( ) >=1 fall(s) in 1 year  **Positive: any of the above** | SPLATT evaluation  Fall prevention  Fracture🡪Osteoporosis evaluation |
| Polypharmacy | ( ) >=8  **Positive: any of the above** | Medication review  Beer’s Criteria |
| Pain | ( ) Pain scale＿＿＿/10　　Location＿＿＿＿  **Positive: Pain scale>=3** | Pain management |
| Pressure sore | ( ) Location＿＿＿  　Grade＿  **Positive: any of the above** | Wound care and monitor  Caregiver education |
| Incontinence | ( ) Urinary  ( ) Fecal  **Positive: any of the above** | Cause evaluation and management  Nonpharmacologic/pharmacologic management |
| Iatrogenesis/tubes | ( ) NG ( ) Foley  ( ) Tracheostomy  ( ) Restraint  ( ) Others＿＿＿  **Positive: any of the above** | Remove unnecessary tubes/Restraint  Education tube care |
| Nutrition | ( ) BMI<=18, ( ) BMI>=27  ( ) Body weight loss 5% in 1 month / 10% in 6 months  ( ) Albumin less than 3.5 g/dl  ( ) Dysphagia / poor intake or diarrhea >= 3 days  **Positive: any of the above** | MNA evaluation  Swallowing test/ST consultation  Diet component modification and education  Dietitian consultation |
| High health care utilization | ( ) Admission >=2 /year  ( ) ER visits >=2 /year  **Positive: any of the above** | Cause analysis/Risk survey/Medical care planning  Caregiver education |
| DNR | ( ) Need to be discussed  ( ) Accepted  **Positive: any of the above** | Family meeting  Hospice consultation |
| Caregiver issue | Main caregiver/Decision maker ________  ( ) Caregiver burden  **Positive: any of the above** | Resource supplement  Social worker consultation |
| Socioeconomic issues | ( ) Liver alone  ( ) Economic problems  **Positive: any of the above** | Caregiver arrangement  Social worker consultation |

Abbreviations: CGS=Comprehensive geriatric screen; GDS=Geriatric Depression Scale; MMSE= Mini-Mental State Examination; ADL=activities of daily living; IADL= Instrumental activities of daily living; SPLATT= Symptoms, Previous falls, Location, Activity, Time, and Trauma; NG= nasogastric: BMI=body mass index; MNA=mini nutritional assessment; ER:emergency room; DNR: Do not resuscitate; ST: speech therapy; PT: physical therapy; OT: occupational therapy.
