# Supplementary material for: The impact of early comprehensive geriatric screening on the readmission rate in an acute geriatric ward: a quasi-experimental study
Source: BMC Geriatr. 2019 Oct 24;19:285. doi: 10.1186/s12877-019-1312-y (PMC6813968; doi:10.1186/s12877-019-1312-y)
Supplement: Supplementary file 2 — Additional file 2: Table S2. Comparison of the readmission vs Non-readmission group. The characteristics of the patients who were readmitted within 30-days compared with those who were not. [file 12877_2019_1312_MOESM2_ESM.docx]

Additional file 2: TableS2 Comparison of Re-admission vs Non-Readmission Group

|  | Readmission group  [N=107] | Non-Readmission group  [N=653] | p value |
| --- | --- | --- | --- |
| Men, no.(%) | 64 (59.8%) | 330 (50.5%) | 0.07 |
| Age, years, mean (SD) | 82.6±7.8 | 81.9±8.4 | 0.41 |
| Weight, Kg, mean (SD) | 56.9±11.1  [N=97] | 58.2±12.9  [N=593] | 0.36 |
| Marital status |  |  |  |
| Live with spouse | 67 (62.6%) | 362 (55.4%) | 0.17 |
| Divorced, widow, or single | 40 (37.4%) | 291 (44.5%) |  |
| Education |  |  |  |
| College/University and above | 12 (11.3%) | 79 (12.1%) | 0.82 |
| Below senior high school | 94 (88.7%) | 574 (87.9%) |  |
| Smoking experience | 21 (19.6%) | 105 (16.1%) | 0.36 |
| Active smoker | 2 (1.9%) | 14 (2.1%) | 0.85 |
| Length of stay ≥10 days | 67 (62.6) | 327 (50.1) | 0.02 |
| Comorbidities, no.(%) |  |  |  |
| Hypertension | 78 (72.9%) | 474 (72.6%) | 0.94 |
| Diabetes mellitus | 42 (39.3%) | 236 (36.1%) | 0.54 |
| Stroke history | 47 (43.9%) | 234 (35.8%) | 0.11 |
| Coronary artery disease | 25 (23.4%) | 128 (19.6%) | 0.37 |
| Chronic obstructive pulmonary disease | 17 (15.9%) | 96 (14.7%) | 0.75 |
| Congestive heart failure | 28 (26.2%) | 110 (16.9%) | 0.02 |
| Atrial fibrillation | 21 (19.6%) | 79 (12.1%) | 0.03 |
| Cancer without metastasis | 25 (23.4%) | 119 (18.2%) | 0.21 |
| Anemia | 18 (16.8%) | 89 (13.6%) | 0.38 |
| Hip fracture | 6 (5.6%) | 39 (6.0%) | 0.88 |
| Dementia | 30 (28.0) | 176 (27.0%) | 0.82 |
| Depression | 8 (7.5%) | 74 (11.4%) | 0.22 |
| History of myocardial infarction | 3 (2.8%) | 13 (2.0%) | 0.48 |
| Peripheral artery disease | 11 (10.3%) | 50 (7.7%) | 0.35 |
| History of peptic ulcer | 27 (25.2%) | 109 (16.7%) | 0.03 |
| Chronic liver disease | 15 (14.0%) | 53 (8.2%) | 0.05 |
| Connective tissue disease | 1 (0.9%) | 10 (1.5%) | 1.0 |
| Chronic kidney disease | 26 (24.3%) | 94 (14.4%) | 0.009 |
| Tumor history | 28 (18.7%) | 104 (16.0%) | 0.48 |
| Metastatic cancer | 7 (6.5%) | 10 (1.5%) | 0.001 |
| Osteoporosis | 17 (15.9%) | 109 (16.7%) | 0.84 |
| Vertebral fracture | 11 (10.3%) | 78 (11.9%) | 0.62 |
| Parkinson's disease | 13 (12.2%) | 64 (9.8%) | 0.46 |
| Charlson comorbidity index ≥ 3 | 69 (64.5%) | 313 (47.9%) | 0.002 |
| Tube use | 38 (35.5%) | 213 (32.6%) | 0.56 |
| Nasogastric tube |  |  |  |
| Foley urinary catheter |  |  |  |
| Tracheostomy |  |  |  |
| Number of discharge medications (± standard deviation) | 6.4 (3.5) | 6.7 (3.4) | 0.25 |
| Barthel index score at admission ≤ 35 | 60 (56.1%) | 283 (43.3%) | 0.01 |
| No. of admission^*^, no.(%) |  |  | <0.001 |
| 0 | 53 (49.5) | 399 (61.1) |  |
| 1-3 | 43 (40.2) | 234 (35.8) |  |
| 4+ | 11 (10.3) | 20 (3.1) |  |
|  |  |  |  |
