# Supplementary material for: The impact of early comprehensive geriatric screening on the readmission rate in an acute geriatric ward: a quasi-experimental study
Source: BMC Geriatr. 2019 Oct 24;19:285. doi: 10.1186/s12877-019-1312-y (PMC6813968; doi:10.1186/s12877-019-1312-y)
Supplement: Supplementary file 3 — Additional file 3: Table S3. Difference of criteria between historical control group and CGS group. The same and difference criteria between historical control group and CGS group (inclusion criteria, exclusion criteria, CGS and CGA). [file 12877_2019_1312_MOESM3_ESM.docx]

Additional file 3: Table S3. Difference of criteria between historical control group and CGS group:

|  |  | Historical comparison group | CGS (early screen) group |
| --- | --- | --- | --- |
| Inclusion criteria | same | 1. who admitted to acute geriatric ward  2. were 65 years or older at the admission | 1.who admitted to acute geriatric ward  2.were 65 years or older at the admission |
|  | different | 1.Admission from July 2011 to June 2012  2.Discharged alive | 1.Admission from June 2013 to Dec 2013 |
| Exclusion criteria | same | Transfer to other medical department (n=12) | Transfer to other medical department (n=13) |
|  | different | Loss follow up after discharge (N=4) | Died prior to discharge (n=16) |
| CGS | different | Not performed | Performed within 48 hr after admission |
| CGA | different | 1.Performed in selected frailty patients.  2.Time point: usually in the middle or late stage of admission course  3. Proportion of patients receiving CGA: low (about 10% of admission patients) | 1.CGA and intervention if CGS showed positive results.  2.Time point: early, after positive results.  3. Proportion of patients receiving CGA: high (all the patients with need) |
